# Evaluation of a novel rapid genomic test including polygenic risk scores for the diagnosis and management of familial hypercholesterolaemia

**DOI:** 10.21542/gcsp.2021.31

**Published:** 2021-12-31

**Authors:** Emma Neves, Tina Khan, Maggie Williams, Marta Carrera, Winston Banya, Ramon Brugada, Carles Ferrer, Deborah J Morris-Rosendahl, Mahmoud Barbir

**Affiliations:** 1 Familial Hypercholesterolaemia Service, Royal Brompton & Harefield Hospital, Part of Guys and St Thomas NHS Foundation Trust, Harefield, UK; 2 Bristol Genetics Laboratory, Southmead Hospital, North Bristol NHS Trust, UK; 3 Cardiovascular Genetics Center and Clinical Diagnostic Laboratory, Institut d’Investigació Biomèdica Girona –IDIBGI, University of Girona-IDIBGI, Girona, Spain; 4 GENinCode Plc (“GENinCode”), Oxford Science Park, John Eccles House, Robert Robinson Avenue, Oxford, UK; 5 Clinical Genetics and Genomics Laboratory, Royal Brompton Hospital, Guy’s and St. Thomas’s NHS Foundation Trust, London, UK

## Abstract

**Introduction**: Familial hypercholesterolaemia (FH) is a common autosomal dominant genetic condition, characterised by elevated LDL cholesterol (LDL-C), leading to premature cardiovascular disease (CVD). Early and accurate diagnosis, with implementation of preventative therapies, has a major impact on reducing premature CVD, morbidity and mortality. Genetic testing is recommended to confirm clinical diagnosis in the proband and enable cascade testing in relatives. There is growing evidence that the risk of CVD conferred by hypercholesterolaemia depends not only on monogenic causes but also on polygenic factors. GENinCode has developed a novel genomic testing system (Lipid inCode^®^) which we have assessed against an accredited National Health Service (NHS UK) genetic screening service in order to validate its diagnostic and clinical utility.

**Methods**: DNA samples from 40 index cases who had been referred for FH testing in an ISO15189-accredited NHS genetic screening service, were retrospectively tested using the Lipid inCode^®^ assay. The results were compared with those from NHS testing.

**Results**: There was absolute concordance in variant detection between both diagnostic tests for monogenic and polygenic FH, the only difference being in the interpretation and classification of DNA variants based on ACMG guidelines, which did not differ by more than one classification class.  The Lipid inCode^®^ test was equivalent to the NHS test in providing comprehensive genetic analysis that included the assessment of both monogenic (FH) and polygenic determinants of blood cholesterol and including a pharmacogenomic assessment of predisposition to statin-related myopathy.

**Conclusion**: The Lipid inCode^®^ diagnostic test can be undertaken with rapid turnaround and gave the same results as those reported by standard NHS genetic laboratory testing. In addition to assessment of monogenic FH, the Lipid inCode^®^ assay provides additional genetic data, such as polygenic factors contributing to hypercholesterolaemia, a polygenic risk score (PRS) for coronary artery disease (CAD), pharmacogenomic testing for statin myopathy, and genetic predisposition to raised Lp(a).

## Introduction

Familial hypercholesterolaemia (FH) is characterised by elevated LDL cholesterol (LDL-C) which deposits in blood vessels leading to premature cardiovascular disease (CVD) if untreated^1^. It is one of the most common autosomal dominant inherited disorders, with a prevalence of 1 in 200–300 in the general population^2–5^. The initial clinical diagnosis of FH is based on clinical and analytical criteria, for which there are different recommendations and guidelines. The ESC recommends the use of the Dutch Lipid Clinic Network (DLCN)^6^, whereas in the UK, the National Institute for Health and Care Excellence (NICE) recommends the use of the Simon Broome (SB) criteria^7^.

Genetic testing is recommended to confirm a clinical diagnosis and provides a cost-effective method of cascade testing in families^8^. Despite international recommendations, however, FH is generally underdiagnosed and undertreated due to a lack of systematic methods to identify individuals with suspected FH, and limited uptake of cascade/ familial testing^9^.

Monogenic autosomal dominant FH is mainly caused by pathogenic variants in the LDL receptor gene (*LDLR*)^1^ and less frequently in the apolipoprotein B (*APOB*)^10–12^, lipoprotein E (*APOE*) (Bea AM Atherosclerosis 2019)^13^ and proprotein convertase subtilisin/kexin type 9 (*PCSK9*)^14^ genes. Even more rarely, autosomal recessive FH may be caused by variants in the LDLR adaptor protein 1 gene (*LDLRAP1*)^15^. However, there is growing evidence that the risk of CVD conferred by hypercholesterolaemia depends also on polygenic background^16,17^. Talmud et al.^16^ reported on the use of a weighted 12-SNP score, which is based on common DNA variants that collectively are associated with raised LDL-C levels and comprise a polygenic risk score for polygenic FH. Therefore, there is growing evidence that the contribution of genetic factors to FH is cumulative^18^.

Early and accurate diagnosis of affected patients and implementation of preventative therapies will have a major impact on reducing CVD, morbidity and mortality. In order to validate its diagnostic and clinical utility, we have evaluated GENinCode’s genomic assay for FH, Lipid inCode^®^, which assesses both monogenic and polygenic FH, and provides additional information such as pharmacogenomic predisposition to the Simvastatin-related myopathy, for implementation in genetic testing for FH. We have compared the results obtained with this assay to those obtained in an ISO15189-accredited National Health Service (NHS UK) FH genetic screening service, which offers a comparative assay.

## Methods

This was a retrospective study based on 40 FH patient samples from index cases who had previously been referred for FH testing in an ISO15189-accredited NHS genetic screening service.

The study was registered as a local audit by our Trust quality and safety department in accordance with local guidelines.

### Patients

A retrospective cohort of 40 FH cases (21 female, 19 male), aged 11 to 72 years, with a clinical diagnosis of FH based on Simon Broome criteria and previous genetic testing performed with the most comprehensive test available in the UK to date (referred to as the NHS assay), was selected for testing using the Lipid inCode^®^ assay. The baseline characteristics of the patients are included in Table 1. All patients had previous measurement of lipid levels and a clinical assessment.

#### Genetic analysis

##### NHS assay

DNA was extracted from EDTA blood samples and processed on a 6-gene (*APOB* (NM_000384.2), *LDLR* (NM_000527.4), *PCSK9* (NM_174936.3), *LDLRAP1* (NM_015627.2), *APOE* (NM_000041.2) and *STAP1* (NM_012108.3) custom-designed panel using the HaloPlex Target Enrichment System kit (Agilent, design 04818-1434990090, version 3) and sequenced using a NextSeq (Illumina) next generation sequencing (NGS) platform. The entire coding region of the genes was targeted, including the promoters and putative branch sites and splice sites (-50/+50 of intronic flanking sequence). Sequence analysis was performed using an open source in-house pipeline, including BWA for alignment to the reference sequence and GATK for variant calling. Variants were annotated using Annovar, with hg19 human genome as a reference. Variant filtering was performed used Geneticist Assistant (Soft Genetics). The sensitivity and specificity of this assay for SNV detection was 100% and 99.9% respectively. The sensitivity and specificity for INDEL detection was 97.73% and 100% respectively. More than 99% of the targeted regions were covered to a minimum of 30x. Copy number variations in the *LDLR* gene were detected by read depth analysis using a custom CNV-detection tool. Positive mutations were confirmed by Multiplex Ligation-Dependent Probe Amplification (MLPA Beckman-Coulter CEQ 8000) using the SALSA PO62-D2 kit (MRC-Holland), containing probes for each of the 18 *LDLR* exons. Variant interpretation and classification was performed according to ACMG guidelines^19^.

The panel also contained two pharmacogenomic SNPs for statin myopathy in the *SLCO1B1* gene (NM_006446.4; rs4149056 c.521T>C and rs2306283 c.388A>G) and the following 12 LDL-C raising SNPs: *PCSK9* (NM_174936.3, rs2479409), *CELSR2* (NM_001408.2, rs629301), *APOB* (NM_000384.2, rs1367117), *ABCG8* (NM_022437.2, rs4299376), *SLC22A1* (NM_003057.2, rs1564348), *HFE* (NM_000410.3, rs1800562), *MYLIP* (NM_013262.3, rs3757354), *ST3GAL4* (NM_006278.2, rs11220462), *NYNRIN* (NM_025081.2, rs8017377), *LDLR* (NM_000527.4, rs6511720), *APOE* (NM_000041.2, rs429358), *APOE* (NM_000041.2, rs7412).

##### LIPID inCode^®^ assay

The LIPID inCode^®^ assay (GENinCode, Barcelona, Spain) is a targeted sequencing-based assay using the SureSelect QXT chemistry for library preparation (Agilent, Santa Clara, USA). The panel includes the *STAP1* (chr4; NM_012108.3; NP_036240.1), and the *LIPA* gene for lysosomal acid lipase deficiency (LALD) in addition to the *LDLR*, *APOB*, *PCSK9, APOE* and *LDLRAP1* genes. Targeted regions included the coding regions of all genes and promoters, including putative branch sites and splice sites (-25/+25 intronic flanking sequence). Sequencing was performed on an Illumina MiSeq and analysed using an internally developed bioinformatic pipeline including BWA to hg19 reference sequence, Samtools and in-house software (Gendicall 3.0, GENinCode). CNV calling was performed for *LDLR*, using in-house validated software and detected CNVs were confirmed by MLPA as described above for the NHS assay. Variant filtering was based on population frequency and variant classification was made based on a proprietary FH database and the American College of Medical Genetics and Genomics (ACMG) guidelines^19^. The sensitivity and specificity of this assay for SNV detection is 99.9%. Targeted regions were 99.9% covered to a minimum of 30x.

**Figure 1.**
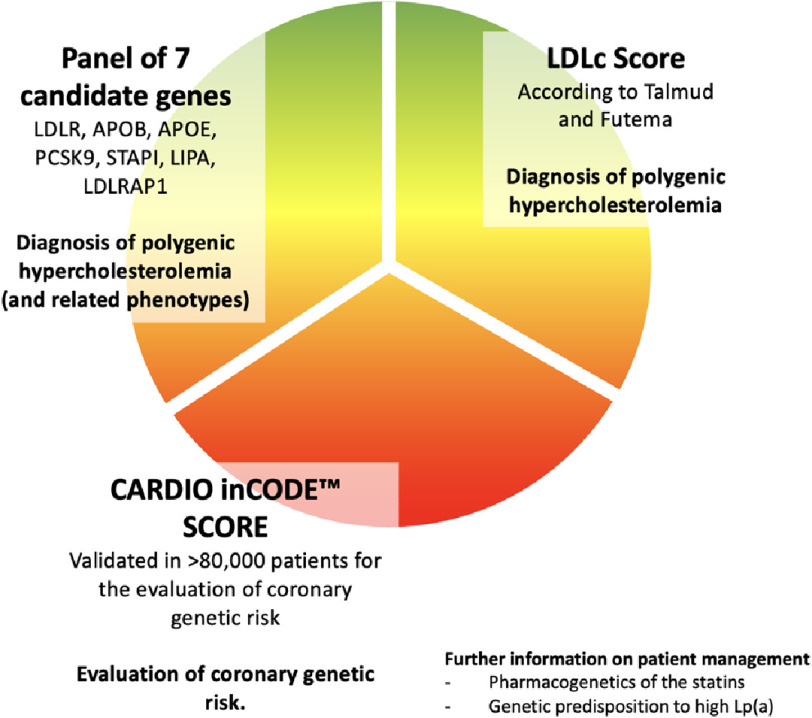
Monogenic diagnosis with polygenic risk stratification.

In addition to the single *SLCO1B1* rs4149056 SNP and the same 12 SNPs for polygenic risk, the assay also includes SNPs in the *HMGCR* (rs17244841) and *ABCB1* (rs2032582) genes for lower response to Simvastatin treatment, and *LPA* rs10455872 and rs3798220 to detect predisposition to elevated Lp(a) levels^20,21^. The LIPID inCode^®^ assay included an additional polygenic risk score (PRS) for coronary artery disease (CAD) (Cardio inCode^®^ Score or CiC)^22^ (Figure 1) comprising 11 SNPs associated with CAD risk independent of cholesterol levels and other classic cardiovascular risk factors screened in the panel: *LPA* (rs10455872), *ALOX5AP* (s10507391, rs17222842 and rs9315051), *PHACTR1* (rs12526453), *CDKN2A/B* (rs1333049), *MIA3* (rs17465637), *WDR12* (rs6725887), *MRAS* (rs9818870), *CXCL12* (rs501120) and *SLC5A3/KCNE2* (rs9982601). SNP scores in quintile 1 indicate low coronary genetic risk; SNP scores in quintile 5 indicate high coronary genetic risk; and SNP scores in quintiles 2 to 4 indicate intermediate coronary genetic risk. The results of this score together with the results of genetic predisposition to elevated Lp(a) levels were not included in this study, as they could not be compared to results of the NHS assay.

#### Comparative study

The Lipid inCode^®^ assay was used for genetic diagnostic testing on the same 40 DNA samples previously tested using the NHS assay. Polygenic risk scores (PRS) for hypercholesterolaemia for both assays were calculated based on the genotypes of the variants and applying the weighting for each SNP as described in the equation by Talmud et al.^16^ and Futema et al.^23^.

The differences between the two tests are summarised in Table 2.

**Table 1:** Patient baseline characteristics and the results of genetic testing. Data are mean for age, with SD in parentheses) and percentage in parentheses for all other entries.

**Variable**	**All subjects**	**Genetically confirmed monogenic FH**	**Polygenic FH**
**No. of patients**	40	25	15
**Mean Age**	45.8 (SD 13.2)	44.5 (SD 13.4)	47.9 (SD 12.9)
**Gender (Male)**	19/40 (47.5%)	12/25 (48.0%)	7/15 (46.87%)
**Ethnicity: Caucasian** **Asian** **Mixed**	32 (80.0%) 7 (17.5%) 1 (2.5%)	19 (76.0%) 5 (20.0%) 1 (4.0%)	13 (86. 7%) 2 (13.3%) 0

**Table 2:** Content and differences between the two assays.

**Genetic analysis**	**FH LIPID inCode^®^ test**	**FH NHS test**
**Sequence analysis**	7 genes: *LDLR, APOB, PCSK9, APOE, STAP1, LDLRAP1* and *LIPA*	6 genes: *LDLR, APOB, PCSK9, APOE, STAP1* and *LDLRAP1*
**LDLR dosage analysis**	MLPA	MLPA
**LDL-C SNP score**	12-SNP score	12-SNP score
**CAD PRS**	11-SNP score (CiC score)	–
**Pharmacogenetics**	Simvastatin-induced myopathy: SLCO1B1 rs4149056	Simvastatin-induced myopathy: SLCO1B1 rs4149056 and rs2306283
	Lower response to Simvastatin treatment: *HMGCR* rs17244841 and *ABCB1* rs2032582	–
**Predisposition to elevated Lp(a) levels**	*LPA*rs10455872 and rs3798220	–

#### Statistical analysis

The probability of polygenic hypercholesterolaemia was based on the genotypes of the 12 known LDL-C-raising SNPs and applying the weighting and equation described by Talmud et al.^16^. In the NHS assay, a SNP score decile of 1–3 was considered low likelihood of polygenic aetiology, a score decile of 6–10 as a high likelihood of polygenic aetiology, and a SNP score decile of 4–5 as average likelihood of polygenic aetiology. For the LipInCode assay, LDL-C polygenic risk score values equal to or above than 1.09 indicate a high probability of polygenic hypercholesterolemia; values equal to or below than 0.73 indicate a low probability of polygenic hypercholesterolemia and scores between 0.73 and 1.09 were considered to reflect intermediate probability. Fisher’s exact test was used to test for significance in the distribution of PRS in patients with and without a monogenic cause of their FH.

## Results

DNA samples from 40 patients with a clinical diagnosis of FH were tested in two different genetic testing regimes, in order to assess concordance, sensitivity and specificity of the results of the two tests. Genetic analysis via Lipid inCode^®^ diagnostic testing was completed over a period of 4 weeks, as compared to six to eight weeks for the NHS genetic screening service, in line with national guidelines.

Details of the patients included in the study and the numbers of patients found to have a monogenic cause or polygenic risk for their FH are provided in Table 1.

### Monogenic FH

All patients were tested by NGS for potentially pathogenic variants in the *LDLR, APOB, PCSK9, APOE* and *LDLRAP1* genes. In addition to those genes, the GENinCode assay also tested for pathogenic variants in the *LIPA and STAP1* genes. Although present in the NHS assay, *STAP1* was not included in analysis. Twenty-five patients had been selected as having a monogenic cause to their FH and the remaining 15 patients had no potentially pathogenic variants (i.e., no pathogenic, likely pathogenic or variants of uncertain significance, VUS) reported in any of the genes analysed. There was 100% concordance between the tests in variant detection, with all potentially pathogenic variants recorded in reports being identical. All variants were classified according to ACMG guidelines, however there were some differences in variant classification between the two laboratories performing the tests, but no differences that were greater than a single class difference in pathogenicity. Variant classifications for 16 of 25 results were identical (13 pathogenic and 3 likely pathogenic). There were differences in variant classification for the remaining 9 cases, with 6 of these being between the classifications pathogenic and likely pathogenic and the remaining 3 between likely pathogenic and VUS. All variants reported, together with their classification, are in Supplementary Table 1. No variants were reported in either the *LIPA* or *STAP1* genes.

Eight patients, 6 of whom had a monogenic cause of FH as above, were reported to be heterozygous for the *LPA* SNP rs10455872, and therefore a predisposition to elevated Lp(a) levels was detected. The remaining two patients heterozygous for the LPA rs10455872 SNP both had polygenic FH.

One patient had a very high CAD PRS value (in the upper range of Q5), which confers a CVD risk equal to that of a heterozygous FH variant. Interestingly, this patient also had a monogenic cause of FH. Furthermore, 16 patients had CAD PRS values in quintile 5, indicating a high coronary genetic risk; 11 of these patients also had a monogenic cause of FH.

### Polygenic risk score assessment for hypercholesterolaemia

There was 100% concordance between the PRS values calculated in each of the laboratories (Supplementary Table 1).

Overall, 23 patients had a high likelihood of having a polygenic cause of their FH, 7 had an intermediate likelihood and 10 had a low risk of a polygenic cause. Of the 23 patients with a high polygenic risk, 14 had a monogenic FH cause in addition to the polygenic risk, as did 4 of the 7 patients with an intermediate polygenic risk. Of the 10 patients with a low polygenic risk, 7 had a monogenic cause and the remaining three therefore have no known genetic cause of their FH. The distribution of patients within each of these categories (i.e., monogenic cause vs no monogenic cause) with respect to their polygenic risk scores, was not significantly different, although the calculation is based on a very small sample size.

The Lipid inCode^®^ assay used more stringent criteria to diagnose polygenic hypercholesterolaemia (PH). LDL-C scores equal to or above 1.09 (score decile 9 or above) indicated a high probability of PH. In contrast, values equal to or below 0.73 (score decile 2 or below) indicate a low probability of PH. Overall, 9 patients had a high likelihood of having a polygenic cause of their hypercholesterolaemia. Of those 9 patients, 5 had a monogenic FH cause in addition to the polygenic risk.

### Statin myopathy

The NHS assay tested for the *SCLO1B1* SNPs rs4149056 and rs2306283 to check for predisposition to statin-related myopathy. The Lipid inCode^®^ assay included the *SCLO1B1* SNP rs4149056 SNP, as well as two additional SNPs, *HMGCR* rs17244841 and *ABCB1* rs2032582, which indicate the likelihood of a lower response to Simvastatin treatment. The results on the *SCLO1B1* rs4149056 SNP were 100% concordant between the assays, with 4 patients with monogenic FH and 2 patients with high PRS for hypercholesterolaemia being heterozygous for the SNP. A single patient with monogenic FH was homozygous for the C allele. The clinical utility of these variants assessing statin-related myopathy and statin response may be limited, as they are based on response to Simvastatin which is no longer the preferred choice of statin therapy in the UK; whereby the majority of adult patients are treated with higher intensity statins such as Atorvastatin or Rosuvastatin.

## Discussion

Despite widespread recognition of the importance of early detection and diagnosis of FH, it remains underdiagnosed and undertreated globally^24–26^. In particular, the offer of genetic testing for FH is under-utilised and there is variation in the availability, targets and quality of different genetic tests offered internationally. The need for a high quality, comprehensive assay with a fast turnaround time is therefore substantial, hence our comparative evaluation of the Lipid inCode^®^ test as a possible solution to addressing this undermet need.

The ability to risk-stratify individuals with FH is essential in order to determine which individuals require intensive therapy, as well as to direct further screening of relatives^27^. Although FH can be suspected clinically, the advantages of genetic testing include the ability to identify affected individuals who do not meet specific cholesterol-level thresholds, as well as facilitating identification of asymptomatic and presymptomatic relatives who may be at risk for a specific familial disease-causing variant via cascade screening^28,29^. It is well known that the presence of a monogenic cause of FH is associated with a significantly greater risk of premature cardiovascular disease compared to those individuals without a monogenic cause of their FH^27^. In fact, knowledge of an individual’s genotype provides important information about cardiovascular disease risk that is independent of LDL-C levels^27^. The 25 patients selected for this study had a wide range of potentially pathogenic variants in three of the five genes initially tested (viz *APOB*, *LDLR* and *PSCK9*), these being the three genes most commonly harbouring pathogenic FH-causing variants. The variants detected ranged from single nucleotide, copy number and splice site variants, all of which were detected by the Lipid inCode^®^ assay. There was 100% concordance between the two assays in variant detection with minor discordances in variant interpretation, either between the classification of VUS vs likely pathogenic, or between likely pathogenic and pathogenic. Differences in the interpretation of potentially pathogenic variants is well-known^30,31^ and is mostly attributable to Clinical Scientist interpretation of the ACMG guidelines, as well as previous findings of particular variants in other patients within diagnostic laboratories. The interpretation and classification of DNA variants remains one of the greatest challenges for clinical genetic diagnostics. The international standardisation of assays and interpretation of guidelines, as well as availability of large variant databases including variant classifications, will all contribute to addressing this in the future.

The *STAP1* gene was originally included in both assays due to the report by Fouchier et al.^32^ documenting its association with FH. This gene-disease association has since been questioned^33–35^ and no potentially pathogenic variants in *STAP1* were identified in this study.

**Figure 2.**
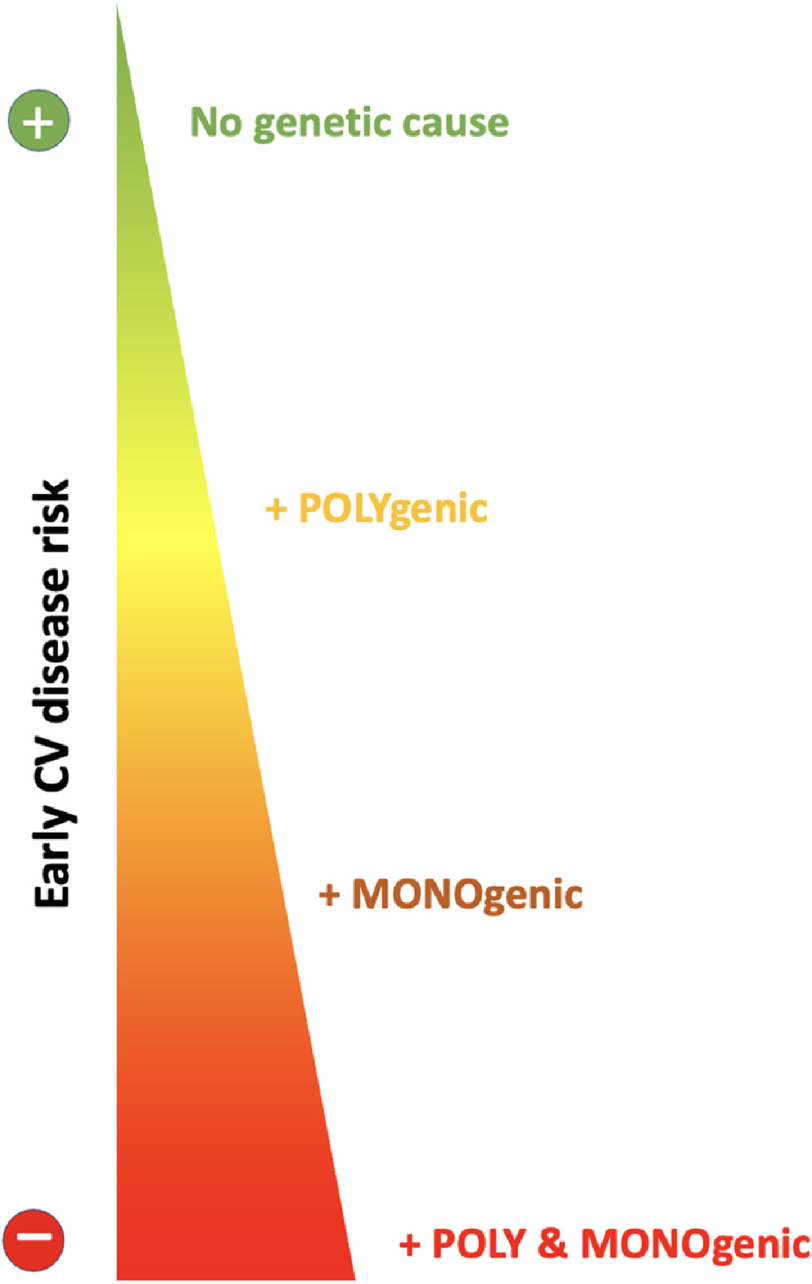
CVD genetic risk stratification.

A polygenic cause of FH, as identified by the polygenic risk score for hypercholesterolaemia, can be identified in 20% to 30% of patients with clinical FH^16,17,36^. Patients without monogenic FH and a high LDL-C polygenic risk score (>80th percentile) have significantly higher baseline LDL-C levels and a higher CVD risk than patients with a lower (<80th percentile) PRS^18,27^. Despite widespread acceptance of the validity of the LDL-C polygenic risk score for the assessment of a polygenic cause of FH^26^, the inclusion of PRS in clinical diagnostic tests for FH is still uncommon. FH will nevertheless serve as a paradigm for the use of PRS in genetic testing, not only as a complementary diagnostic test for polygenic causes, but also as a possible explanation for modifying effects on monogenic disease^27^ (Figure 2). Within this cohort, of the 23 patients with a high polygenic risk, 14 concurrently had a monogenic FH cause in addition to the polygenic risk illustrating that this combination occurs frequently, and in such patients may represent a higher cardiovascular risk profile compared to patients with monogenic FH in the absence of additional high polygenic risk. Recent evidence points to the fact that LDL-C PRS may have added value in both monogenic and polygenic forms of FH. In a meta-analysis of over 1000 FH mutation-positive individuals from three different cohorts (including UK BioBank), those with an LDL-SNP score above the 80th percentile had a 48% higher risk of atherosclerotic CVD^18^. This risk was in part but not fully explained by adjustment for LDL-C.

Overall, the published studies support the utility of reporting the score in all patients. Both assays ascertained in this study calculated the PRS for all patients, although the NHS test routinely only reports PRS where a monogenic cause has not been found. This is not the case with the Lipid inCode^®^ assay, that reports the LDL-C score independently of the identification of a monogenic cause for FH. Our data demonstrates that omitting polygenic testing of individuals with monogenetic mutations may miss opportunities to further risk stratify patients with particularly high cardiovascular risk with both monogenetic and polygenic aetiology. There is strong clinical utility of the LDL-C PRS in individuals with a clinical diagnosis of FH (a high PRS, in the top 1–2 deciles of the score, and no FH-causing monogenic variant), since these individuals are at a high risk of future CHD and should be considered for more intense lipid lowering therapy, as can be best managed in a lipid clinic. In addition, monogenic FH patients with a PRS score >80th percentile should be considered for more intensive lipid lowering therapy than those with scores <80th percentile (possibly including PCSK9 inhibitors https://www.nice.org.uk/guidance/TA39) to reduce their LDL-C and CVD risk.

Pathogenic variants in the *LIPA* gene are associated with Cholesteryl Ester Storage Disease (CESD) and Wolman disease, both autosomal recessive allelic disorders (OMIM # 278000) associated with reduced activity and genetic defects of lysosomal acid lipase. In addition to the inclusion of the *LIPA* gene in the NGS panel of the Lipid inCode^®^ assay, the test also assesses two SNPs associated with a predisposition to raised levels of Lipoprotein(a)^37^. Heterozygosity for rs10455872 of the *LPA* gene, as detected in 8 of the patients analysed in this study, increases the possibility of having high plasma levels of Lp(a), an independent marker of cardiovascular risk^38^ however we did not observe raised Lp(a) in these patients.

There is wide acceptance of the fact that genetic variation is responsible for interindividual response to statin therapy^39^ and the testing for this response in patients with FH, in order to guide the selection of lipid-lowering therapy, is directly aligned with the principles of personalised medicine. *SLCO1B1* polymorphisms clearly impact the pharmacokinetics of Simvastatin and, to a lesser degree, the pharmacokinetics of other statins^40^. The rs4149056 T>C SNP in *SLCO1B1* increases systemic exposure to Simvastatin and the risk of muscle toxicity^41^. As highlighted earlier, although these statin response polymorphisms serve as an indicator, the clinical application of these polymorphisms may be limited due to the preferential use of higher intensity statins such as Atorvastatin and Rosuvastatin in contemporary practice. Future identification of polymorphisms that are capable of predicting the efficacy and likelihood of intolerance of higher intensity statins are likely to influence and customise therapeutic decisions.

The Cardio inCode^®^ score (CiC score)^22^, a PRS for CAD, was included only in the Lipid inCode^®^ test and is a measure of a patient’s genetic risk for coronary disease independent of other cardiovascular risk factors (including hypercholesterolaemia) and of family history of CHD^22,42,43^. There is evidence that a high CAD PRS can significantly modify the disease phenotype (higher prevalence of CVD events and higher number of events) after adjusting for established predictors of CAD risk, even in the context of a severe monogenic disease such as FH^44,45^. A high CAD PRS has also been shown to be associated with greater subclinical atherosclerosis in FH patients, as assessed by coronary artery calcium score, even after adjusting for the presence of pathogenic FH variants^45^. Although the utility of this additional genetic data in clinical practice should be confirmed with large scale clinical trial data, and is beyond the scope of this study, it may refine CVD risk prediction in FH patients and this could lead to a more personalized approach to patient management and therapy. A high CAD PRS in a patient with FH could serve as a rationale for more aggressive LDL-C lowering, including the addition of a PCSK9 inhibitor^46^. Therefore, this coronary genetic risk score could be considered as an adjunct for risk assessment and stratification at this stage, rather than a surrogate for conventional established methods of clinically assessing cardiovascular risk.

## Conclusion

Several different molecular and biochemical tests are required to interrogate the full range of potential genetic factors underlying FH and early detection is important in order to ensure that patients are offered preventative interventions, both lifestyle modification^42^ and lipid-lowering therapy^25,47^, to reduce the morbidity and mortality associated with this condition. The availability of a comprehensive assay covering all known genetic and pharmacogenomic risk factors for FH, with a fast turnaround time, is therefore key to clinical management of patients and their family members.

This study has demonstrated that the Lipid inCode^®^ diagnostic test is a good comparator to the NHS accredited laboratory genomics test enabling a rapid turnaround with comprehensive genetic analysis that will help to increase diagnostic efficiency and promote timely implementation of clinical management. In addition to conventional assessment of monogenic FH, the Lipid inCode^®^ assay may enhance clinical diagnosis and management by offering additional genetic data such as a polygenic risk score (PRS) for hypercholesterolaemia, pharmacogenomic testing for statin myopathy, and genetic predisposition to raised Lp(a), an important independent cardiovascular risk factor. Lipid inCode^®^ also offers a coronary genetic risk score which is a value that indicates the genetic contribution to coronary risk and is determined by the weighted sum of the influence of each of the genetic variants analysed.

The heterogeneity in CV risk in FH remains inadequately understood, but it is likely to involve genetic variation that affects both LDL-C levels and broader CV risk^46^. Although further assessment of the utility of a CAD PRS requires investigation in prospective cohorts, and is beyond the scope of this study, the field is gradually evolving towards the increased use of molecular genetic information to guide the diagnosis, risk prediction, and management of patients with FH^46^.

## Conflicts of interest

MB is a clinical advisor to GENinCode. DMR is a clinical and scientific advisor to GENinCode. TK, EN and WB have no conflicts of interest to declare. RB is a clinical and scientific advisor to GENinCode. MC and CF are GENinCode employees. MW has no conflicts of interest to declare.

## Acknowledgement

We would like to acknowledge Elizabeth Watson and Francheska Punzal for help in the NHS processing and sending of samples.
